# Spanish-dementia knowledge assessment scale (DKAS-S): psychometric properties and validation

**DOI:** 10.1186/s12877-021-02230-w

**Published:** 2021-05-10

**Authors:** A. Carnes, E. Barallat-Gimeno, A. Galvan, B. Lara, A. Lladó, J. Contador-Muñana, A. Vega-Rodriguez, M. A. Escobar, G. Piñol-Ripoll

**Affiliations:** 1 Unitat Trastorns Cognitius, Clinical Neuroscience Research, Santa Maria University Hospital, Rovira Roure n 44. 25198. IRBLleida, Lleida, Spain; 2 Faculty of Nursing and Phisiotherapy, Universitat de Lleida, IRBLleida, Lleida, Spain; 3 Alzheimer’s Disease and Other Cognitive Disorders Unit, Department of Neurology, Hospital Clínic, Institut d’Investigació Biomèdica August Pi I Sunyer, Barcelona, Spain; 4 University of Salamanca, Valladolid, Spain

**Keywords:** Alzheimer’s disease, Dementia, Knowledge, DKAS, Spanish, Validation studies, Non-professional caregivers, Students

## Abstract

**Background:**

Alzheimer’s disease (AD) is the most frequent cause of cognitive impairment. Community knowledge of the disease has proven to be a very important aspect of the development of interventions and the evaluation of their effectiveness. However, it is necessary to have standardized and recognized tools in different languages. The aim of the current study was to develop a cross-cultural adaptation of the Spanish Dementia Knowledge Assessment Scale (DKAS-S) and to assess their psychometric properties with cohorts of health students and professional and non-professional caregivers of AD patients from several regions of Spain.

**Methods:**

We developed and translated the DKAS into Spanish following the forward-back-forward translation procedure. Then, we performed a cross-sectional study to assess the validity, reliability and feasibility of the DKAS-S. We also performed an analysis to obtain test-retest reliability measures. The study was performed in four medical centres across three regions in Spain. From May to September 2019, we administered the scale to students, professional and non-professional caregivers; including a subgroup of non-professional caregivers of patients with early-onset AD (< 65 years).

**Results:**

Eight hundred forty-six volunteer participants completed the DKAS-S: 233 students (mean age 26.3 ± 9.2 years), 270 professional caregivers (mean age 42.5 ± 11.7 years) and 343 non-professional caregivers of AD patients. (mean age was 56.4 ± 13.16). The DKAS-S showed good internal consistency (Cronbach’s α = 0.819) and good test-retest reliability (time 1: 28.1 ± 8.09 vs time 2: 28.8 ± 7.96; t = − 1.379; *p* = 0.173). Sensitivity to change was also significant in a subgroup of 31 students who received education related to AD and dementias between each administration (time 1: 25.6 ± 6.03) to (time 2: 32.5 ± 7.12; t = − 5.252, *p* = 0.000). The validity of the construct was verified by confirmatory factor analysis, although there were challenges in the inclusion of some items in the original 4 factors.

**Conclusions:**

The 25-item DKAS-S showed good psychometric properties for validity and reliability and the factorial analysis when it was administered to a population of students and professional and non-professional caregivers. It was a useful instrument for measuring levels of knowledge about dementia in Spanish population.

**Supplementary Information:**

The online version contains supplementary material available at 10.1186/s12877-021-02230-w.

## Background

Alzheimer’s disease (AD) is the most frequent cause of cognitive impairment in subjects older than 65 years, representing between 50 and 70% of patients with cognitive impairment [1]. The global prevalence of dementia is increasing, and it will represent an economic, social and health problem of great magnitude in the near future. Therefore, health policies should focus on investigating not only effective pharmacological treatments but also preventive measures and ways we can improve the quality of life of patients and caregivers.

Community knowledge of the disease has proven to be a very important aspect of improving the early detection of dementia [2–5], and it can help to reduce stigma and eliminate social stereotypes [6, 7]. Improving knowledge of dementia management through health education conducted by health professionals can improve clinical and community care in domestic and specialized settings [8]. Conversely, poor knowledge among populations and health professionals can cause a delay in the diagnosis or cause confusion regarding the symptoms, which would lead to an inappropriate diagnosis and management [9, 10].

Therefore, an assessment of one’s knowledge of the concept of dementia and/or AD and its implications will be useful among health professionals, the general population and caregivers, allowing us to discern differences in this knowledge between cohorts responsible for the care and treatment of patients with the disease [11]. This will allow us to develop and evaluate the effectiveness of interventions or to evaluate at the population level whether information campaigns have achieved their established objectives.

To achieve this goal, it is necessary to have standardized and recognized tools that facilitate the collection of such knowledge about dementia to avoid biased and subjective results. These tools must be of good quality with adequate psychometric properties, and they must be adapted to the cultural, linguistic and social characteristics of each community. It is essential that these tools measure baseline knowledge and changes in knowledge following educational interventions.

Until the date of publication of the Dementia Knowledge Assessment Scale (DKAS), there were thirteen scales, but only three with acceptable reliability and validity were available to measure the degree of knowledge about dementias: the *Dementia Quiz* (DQ) [12], the *Knowledge of Aging and Memory Loss and Care* (KAML-C) [13] and the *Alzheimer’s Disease Knowledge Scale* (ADKS) [14]. According to a systematic review, all measures followed the process of developing a standardized scale, and most have acceptable psychometric properties in terms of reliability and validity. However, some of the limitations indicated by the authors are that these scales have limited scope, some are outdated, and the evaluation does not cover different conceptual domains [15]. Thus, Annear and collaborators designed the DKAS with the aim of correcting these limitations. The test-retest reliability values ​​were high, and the internal consistency and preliminary construct, concurrent and factor validity were good [16]. The same authors compared the efficacy of the DKAS with that of the ADKS and concluded that the DKAS had a superior internal consistency, a wider response distribution and a lower ceiling effect than the ADKS, as well as better discrimination between scores obtained before and after respondents received health education on dementia [17]. The scale comprises 25 statements about dementias, and subjects are asked to answer on a Likert scale with five response options: true, probably true, probably false, false, and don’t know. The same authors performed the factor analysis establishing four domains: 1) causes and characteristics (dementia pathology and terminal course), 2) communication and behaviour (how a person with dementia engages with the world, 3) care considerations (dementia symptoms relevant to the provision of care) and 4) health risk and promotion (risk factors and conditions that are associated with or mistaken for dementia) [18].

To our knowledge, there is no Spanish-language scale assessing the degree of dementia knowledge that has been validated with the main target population of these tools, namely, the non-professional caregivers. Previously, the Dementia Knowledge Assessment Tool 2 (DKAT2) was translated into the Spanish language and validated, but only with nursing students and nursing staff from nursing homes [19]. Parra-Anguita et al. developed and validated a scale to measure AD knowledge among both Spanish nursing staff and students (UJA Alzheimer’s Care Scale) but this scale also had not been designed for non-professional caregivers [20]. There has also been another attempt in the Spanish language to determine the usefulness of ADKS among non-professional caregivers, but the tool had relatively low internal consistency. Despite this limitation, the results are consistent with those in the previous literature that the duration of the disease improved the degree of knowledge of the symptoms and the recognition of the illness by the caregivers [21].

The aim of the current study was i) to develop a cross-cultural adaptation of the Spanish Dementia Knowledge Assessment Scale (DKAS-S) and ii) to assess the psychometric properties with cohorts of health students and professional and non-professional caregivers of AD patients from several regions of Spain.

## Methods

### Design

A cross-sectional study was designed to assess the validity, reliability and feasibility of the DKAS-S. We also performed the analysis to obtain the test-retest reliability measures. The study was performed in four medical centres across three regions in Spain.

### Translation and back-translation

The translation of the DKAS into Spanish was carried out using a forward-back-forward translation procedure. Three experts who were bilingual in English and Spanish translated the original version of the DKAS into Spanish. After consensus was reached among researchers and translators, a first version was agreed upon. Three other experts also bilingually performed back translation. Then, a comparison between the original scale and the one from the translation was made to identify discrepancies. Finally, two more experts translated the scale into Spanish again. The final version was obtained through consensus among researchers and a linguist to select the best translations within the cultural context of the tool. They corrected grammatical, linguistic and semantic aspects. It was pretested and further reviewed by a potential group of target populations (30 non-professional caregivers of AD patients from the Memory Unit of Hospital Santa Maria Lleida). The DKAS-S is shown in Table 1.

**Table 1:** Original (English) and Spanish versions of the Dementia Knowledge Assessment Scale

DKAS (item in English)	DKAS-S (item in Spanish)
1. Dementia is a normal part of the ageing process. (FALSE)	1. La demencia es una fase normal del envejecimiento.
2. Alzheimer’s disease is the most common form of dementia. (TRUE)	2. La enfermedad de Alzheimer es la forma más común de demencia.
3. People can recover from the most common forms of dementia. (FALSE)	3. Las personas pueden recuperarse de las formas más comunes de demencia.
4. Dementia does not result from physical changes in the brain. (FALSE)	4. La demencia no es el resultado de cambios físicos en el cerebro.
5. Planning for end of life care is generally not necessary following a diagnosis of dementia. (FALSE)	5. Planificar los cuidados del final de vida generalmente no es necesario después un diagnóstico de demencia.
6. Blood vessel disease (vascular dementia) is the most common form of dementia. (FALSE)	6. La demencia vascular es la forma más común de demencia.
7. Most forms of dementia do not generally shorten a person’s life. (FALSE)	7. Generalmente la mayoría de demencias no acortan la esperanza de vida de una persona.
8. Having high blood pressure increases a person’s risk of developing dementia. (TRUE)	8. Tener una presión arterial alta aumenta el riesgo de tener demencia.
9. Maintaining a healthy lifestyle does not reduce the risk of developing dementia. (FALSE)	9. Mantener un estilo de vida saludable no reduce el riesgo de tener las formas más comunes de demencia.
10. Symptoms of depression can be mistaken for symptoms of dementia. (TRUE)	10. Los síntomas de depresión pueden confundirse con síntomas de demencia.
11. Exercise is generally beneficial for people experiencing dementia. (TRUE)	11. Generalmente el ejercicio físico es beneficioso para personas con demencia.
12. Early diagnosis of dementia does not generally improve quality of life people experiencing the condition. (FALSE)	12. Generalmente el diagnóstico precoz de la demencia no mejora la calidad de vida de los pacientes que tienen la enfermedad.
13. The sudden onset of cognitive problems is characteristic of common forms of dementia. (FALSE)	13. La aparición repentina de problemas cognitivos es típico de las formas más comunes de demencia.
14. It is impossible to communicate with a person who has advanced dementia. (FALSE)	14. Es imposible comunicarse con una persona que tiene una demencia avanzada.
15. A person experiencing advanced dementia will not generally respond to changes in their physical environment. (FALSE)	15. Generalmente una persona con demencia avanzada no responde a los cambios de su entorno.
16. It is important to correct a person with dementia when they are confused. (FALSE)	16. Es importante corregir a una persona con demencia cuando está confundida.
17. People experiencing advanced dementia often communicate through body lenguaje. (TRUE)	17.Generalmente las personas con demencia avanzada se comunican mediante lenguaje corporal.
18. Uncharacteristic behaviours in a person experiencing dementia are generally a response to unmet needs. (TRUE)	18. Generalmente las conductas anormales en personas con demencia responden a necesidades no satisfechas.
19. Medications are the most effective way of treating behavioural symptoms of dementia. (FALSE)	19. La medicación es la forma más efectiva de tratar los síntomas conductuales de las demencias.
20. People experiencing dementia do not generally have problems making decisions. (FALSE)	20. Generalmente las personas con demencia no tienen problemas para tomar decisiones.
21. Movement is generally affected in the later stages of dementia. (TRUE)	21. El movimiento generalmente se ve afectado en las últimas etapas de la demencia.
22. People with advanced dementia may have difficulty speaking. (TRUE)	22. Las personas con demencia avanzada pueden tener dificultades para hablar.
23. People experiencing dementia often have difficulty learning new skills. (TRUE)	23. Las personas con demencia a menudo tienen dificultades para adquirir nuevas habilidades.
24. Difficulty eating and drinking generally occurs in the later stages of dementia. (TRUE)	24. Las dificultades para comer y beber generalmente aparecen en las últimas etapas de la demencia.
25. Daily care for a person with advanced dementia is effective when it focuses on providing comfort. (TRUE)	25. El cuidado diario de una persona con demencia avanzada es efectivo cuando se centra en el confort del paciente.

### Sample and administration

The translated and culturally adapted DKAS-S was used to test its validation and psychometric properties. From May to September 2019, we administered the scale with a cohort of health students (*n* = 233; nursing (*n* = 135) and psychologists (*n* = 98)), professional caregivers (*n* = 270) and non-professional caregivers (*n* = 343) of AD patients from three different regions of Spain (Catalonia (two different hospitals in Lleida and Barcelona), Aragon and Castilla y León). Professional caregivers were recruited from several nursing homes around Lleida County and Aragón. Non-professional caregivers were recruited consecutively during the period of the study from the Unit Memory of Hospital Santa Maria Lleida, and from Alzheimer’s family association of Huesca and Salamanca. We administered the scale consecutively in a subgroup of 32 non-professional caregivers of early-onset AD patients (< 65 years) (EOAD) from Hospital Clinic de Barcelona. Localizations were purposively selected to provide representation from different zones of the country.

Test-retest reliability was explored in a sample of 67 subjects, 30 professional-caregivers, 29 non-professional caregivers of LOAD and 8 non-professional caregivers EOAD that were analysed after 4 weeks from the first evaluation.

Besides, a subgroup of 31 nursing students received education related to AD and dementias for 2 h of class between the first and second administration of the scale.

First, permission from the original author of the DKAS (Dr Annear) was obtained. The Scientific Ethics Committee of the Hospital Universitari Arnau de Vilanova approved both the study and the consent procedure (CEIC 2119). We obtained written informed consent from all the participants before including them. Participation was completely voluntary. After signing the informed consent, participants were given a copy of the scale, which they completed in approximately 15 min. Participants’ anonymity and confidentiality were guaranteed.

### Data availability

The data reported in this manuscript are available within the article and/or its supplementary data. Additional data will be shared by request from any qualified investigator.

### Statistical analysis

Statistical analyses were performed following the steps of the construction of the original scale [14]. First, a descriptive analysis of all the variables of the sample for each group (students, professional caregivers, non-professional caregivers) was performed using comparisons of means and standard deviations for continuous variables and comparisons of proportions for categorical variables. Second, the means of the responses to the final questionnaire of each group were compared using the ANOVA test. Third, psychometric analyses of the scale were performed. Internal consistency analyses of the full scale and each of the subscales that compose it were made using Cronbach’s alpha. Temporal stability was verified by administering the scale to the same group of subjects at two different times (with a month between the two administrations) and analysed using the t-test for paired data. In the same way, sensitivity to change was verified by administering the scale to a group of students on two different occasions, with training related to AD and dementias received between each application of the scale. These results were analysed using the t-test for paired data. Finally, the validity of the construct was verified by confirmatory factor analysis by extraction of the main components and varimax rotation. The statistical study was performed by the SPSS 24.0 program (SPSS, Chicago, IL).

## Results

### Characteristics of participants

In total, 846 volunteer participants completed the DKAS-S (Table 2). The mean age of the non-professional caregivers was 56.4 ± 13.16 years, of whom 60% of the respondents were sons/daughters. In contrast, in the group of patients with EOAD, the majority of the respondents were partners (81%), with a mean age of 61.9 ± 10.9. The mean age of the students was 26.3 ± 9.2.

**Table 2:** Demographic characteristics of Spanish-Dementia Knowledge Assessment Scale (DKAS-S) responders

	Total	Students	Professionals caregivers	Non-professional caregivers	Non-professional caregivers early AD
	***n*** = 846	***n*** = 233	***n*** = 270	***n*** = 311	***N*** = 32
**Female (%)**	77.6%	82.8%	86.3%	69.0%	50.0%
**Mean age (SD)**	43.9 (17.0)	26.3 (9.2)	42.5 (11.7)	56.3 (13.1)	61.9 (10.9)
**Highest education level**					
**Elementary school (%)**	10.8%	0.0%	1.5%	24.7%	25.0%
**High school (%)**	36.4%	49.3%	23.0%	38.8%	34.4%
**University degree (%)**	31.9%	19.3%	43.3%	30.8%	37.5%
**Higher university degree (%)**	20.9%	31.4%	32.2%	5.7%	3.1%
**Occupation**					
**Not working / retired**	14.2%	6.9%	0.4%	29.4%	37.5%
**Studying**	15.2%	54.9%	0.0%	0.0%	0.0%
**Health work**	41.7%	26.6%	97.4%	7.8%	9.4%
**Unrelated health work**	28.9%	11.6%	2.2%	62.8%	53.1%
**Contact with dementia people**					
**No**	35.6%	50.4%	23.3%		
**Family**	39.8%	28.0%	41.1%		
**Other**	24.6%	21.6%	35.6%		
**Family history of cognitive impairment**					
**Yes**	42.1%	41.2%	48.5%	36.7%	46.9%
**Relationship with the patient**					
**Partner**	35.9%			18.5%	81.3%
**Sons**	47.2%			60.1%	12.5%
**Brothers**	2.2%			2.6%	3.1%
**Other**	14.66%			18.84%	3.1%

### Discrimination between groups

**Fig. 1:**
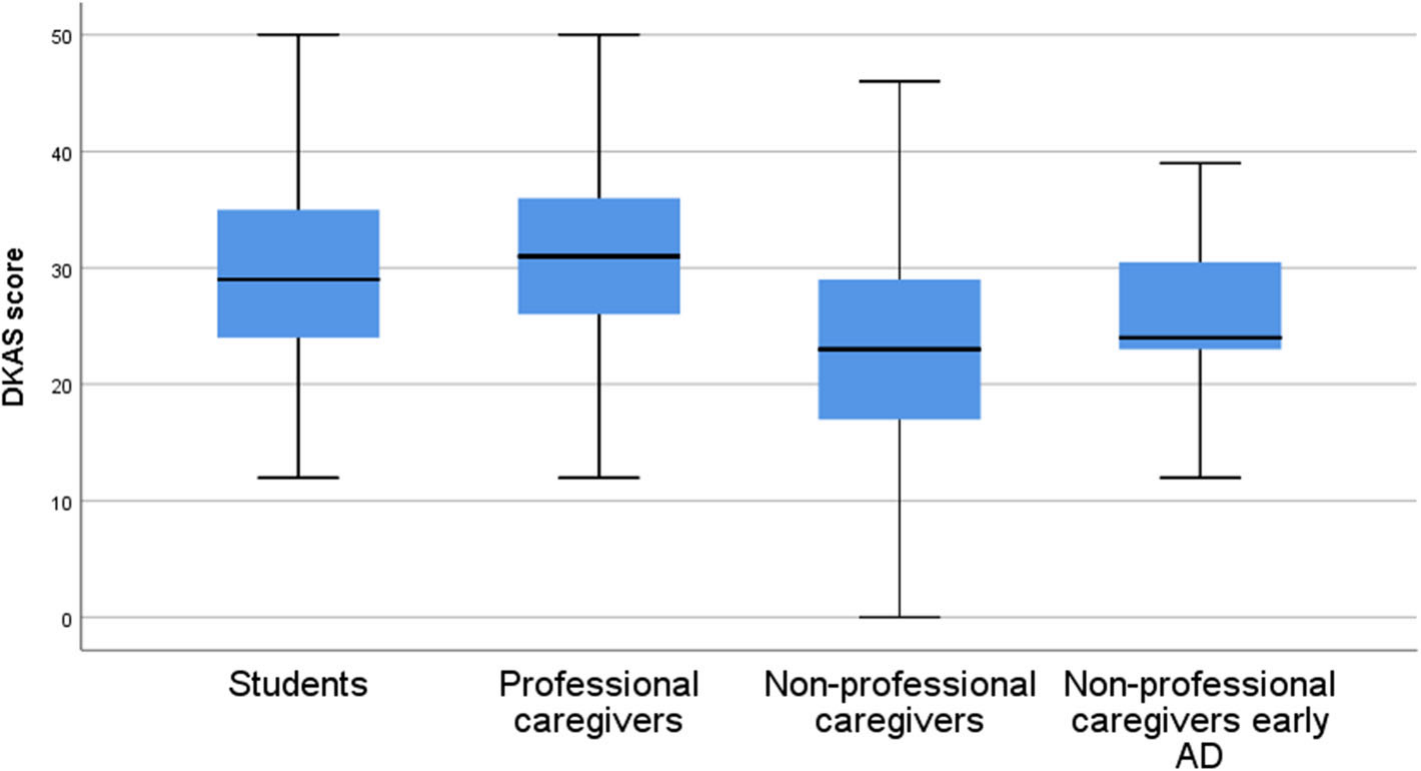
Boxplot of DKAS-S scores among groups

The mean score of all subjects on the scale was 27 points of a total possible score of 50. Professional caregivers (31.28 ± 7.12) scored higher on the scale, followed by students (29.52 ± 7.65), while non-professional caregivers (23.06 ± 8.73) scored the lowest (*p* < 0.000). Comparing non-professional caregivers of EOAD (25.62 ± 7.09) vs late onset (LOAD) (23.06 ± 8.73) we did not find any differences (*p* = 0.18). Despite no statistically significant differences between groups were observed, these results proved that the DKAS-S allows to objectify some differences between groups (Fig. 1).

DKAS-S is divided in four subscales attending knowledge of causes and characteristics, communication and behaviour, care considerations and health risk and promotion. In causes, students (8.81 ± 3.31) scored higher on the subscale, followed by professional caregivers (8.71 ± 2.72), non-professional caregivers of EOAD patients (7.03 ± 2.82) while non-professional caregivers (6.24 ± 3.23) scored the lowest (*p* < 0.000).

In relation to communication and behaviour, professional caregivers (6.07 ± 2.86) scored higher on the subscale, followed by students (5.00 ± 2.33), while non-professional caregivers of LOAD (3.63 ± 2.79) and EOAD (3.28 ± 2.01) scored the lowest (*p* < 0.000). About care, professional caregivers (9.74 ± 2.49) and non-professional caregivers of EOAD (9.53 ± 2.55) scored higher on the subscale, followed by students (8.87 ± 2.59) and non-professional caregivers of LOAD (8.24 ± 3.07) scored the lowest (*p* < 0.000). Finally, the knowledge of risk factor was better in students (6.82 ± 2.45), followed by professional caregivers (6.74 ± 2.53), non-professional caregivers of EOAD (5.78 ± 2.29) and non-professional caregivers of LOAD (4.96 ± 2.52) (*p* < 0.000).

### Internal consistency

The DKAS-S had good internal consistency, with a Cronbach’s alpha coefficient of 0.819, while the scores for each of the subscales were lower and ranged from 0.556 to 0.718, which approached the acceptability criterion of > 0.70 and was consistent with those of other validated scales reported in the health literature [22]. This result indicates that there is an underlying association between each of the subscales within the 25 items of the DKAS-S but that the correlations are not high enough to indicate redundancy or thematic duplication across the four domains.

### Test-retest reliability

Test-retest reliability was explored in a sample of 67 subjects, including professional and non-professional caregivers of LOAD and EOAD non-professional caregivers who had not received dementia education. The results indicated that there was no significant change in the DKAS-S score from time 1 to time 2 (28.1 ± 8.09 vs 28.8 ± 7.96); t = − 1.379; *p* = 0.173), indicating test-retest reliability in this group.

### Sensitivity to change

In a subgroup of 31 nursing students who received education about AD and dementia, there was a statistically significant increase in the dementia knowledge score measured with the DKAS-S from time 1 (25.6 ± 6.03) to time 2 (32.5 ± 7,12); (t = − 5.252, *p* = 0.000). This result reflected a 27% improvement in median scores attributable to the educational intervention.

### Confirmatory factor analysis

The validity of the construct was verified by confirmatory factor analysis (CFA). For most of the items, the eigenvalue was good, approaching the acceptability criterion of > 0.20 (Table 3). However, there were challenges in the inclusion of some items in the original subscale. These items were 8, 10, 13 and 16; three of them belonged to subscale 2 (health risk and promotion). Although items 13 and 16 apparently seemed to be redundant, we tried to remove them, but the CFA did not improve.

**Table 3:** Pattern Matrix for the 25-Item Spanish-Dementia Knowledge Assessment Scale (DKAS-S)

	Subscale 1: causes and characteristics	Subscale 2: health risk and promotion	Subscale 3: communication and behavior	Subscale 4: care considerations
Item 1	0.46			
Item 2	0.42	0.27		0.28
Item 3	0.6			0.25
Item 4	0.44	0.36		
Item 5	0.28	0.63		
Item 6	0.62		0.21	
Item 7	0.38	0.28		
Item 8	0.26		0.42	
Item 9		0.58		
Item 10			0.47	
Item 11		0.31	0.44	0.31
Item 12		0.69		
Item 13	0.45			
Item 14	0.27	0.34	0.43	
Item 15	0.44		0.44	
Item 16	0.48			0.21
Item 17			0.62	0.24
Item 18			0.59	
Item 19	0.39		0.26	
Item 20	0.25	0.41		0.26
Item 21				0.66
Item 22				0.73
Item 23				0.76
Item 24				0.62
Item 25				0.54

## Discussion

The findings of our research demonstrated that the 25-item DKAS-S showed good psychometric properties for validity, reliability and factorial analysis when it was administered to a population of students and professional and non-professional caregivers. The DKAS-S had internal consistency, which indicates that all the items measured the same underlying construct of dementia knowledge. Additionally, the test-retest reliability was confirmed in the three population groups that were analysed, demonstrating that there were no significant differences in the score of the scale after 4 weeks. In contrast, sensitivity to change was proven. The subgroup of students who received an educational training on dementias scored higher on the second administration of the scale after 1 month. The subjects who would be expected to have more knowledge about dementia, such as students and professional caregivers, achieved higher scores on the DKAS-S. Within the group of non-professional caregivers, the relatives of patients with EOAD proved to have better knowledge about dementias than the rest of the non-professional caregivers. Finally, CFA generally showed the same pattern of four factors from the original scale. Although some items did not enter the original subscale (items 8, 10, 13 and 16), they did not change the factor analysis when they were removed, so we did not eliminate them. We interpreted this finding as a possible result of the difficulty in comprehending these questions for some of the subjects, mainly those with a lower previous level of education, and as a possible effect of sample size.

Our results almost reach the levels of psychometric validation of the original scale [15] and exceed those of the validation in Japanese [23], the only one carried out thus far, to our knowledge, with the same scale. The Spanish version of the scale is quite similar to the original, both in structure and item wording. There were no items from the original item that had no semantic equivalence in Spanish. Furthermore, we consulted with a linguistic expert who corrected the grammatical, linguistic and semantic aspects after the translation and back-translation. Therefore, it was possible to maintain the same items and structure and not make major modifications from the original scale. Thus, we were able to maintain the balance of correct and false answers in the questionnaire, which improved the reliability of the responses [11]. In the Japanese adaptation and validation, nine items had to be eliminated because the psychometric properties would otherwise be low if the original 25 items were retained [23]. The administration of DKAS-S does not require the presence of any healthcare professional.

To the best of our knowledge, the DKAS-S is the second major measure of dementia knowledge that has been translated into Spanish and validated in the Spanish population, but it is the only measure that has been subjected to this level of analysis using a large sample, including students and both professional and non-professional caregivers. The first translation and validation attempt used the DKAT2, which showed a lower internal consistency both in the original scale and in the Spanish version than did the DKAS-S, and it was only validated using nursing staff and nursing students [19]. In previous studies using other scales, the validations also included different samples, such as nurses, physicians, psychologists, social workers and medical students [24–27]. ADKS had been used to evaluated the knowledge on AD among community pharmacists and general practitioners [28] and in non-professional caregivers in Spain [21], but one analysis of its psychometric properties in a Spanish version showed that ADKS does not present a unidimensional structure, although its independent items together provide a comprehensive spectrum of information regarding AD knowledge [29]. Recently, Parra-Anguita et al. developed UJA Alzheimer’s Care Scale for measuring knowledge of Alzheimer’s disease and dementia care among nursing professionals or nursing students. The initial validation study obtained good psychometric properties concerning validity, reliability and strong intraclass correlation coefficient with DKAT2 [20]. However, thus far, the DKAS is the only scale that has been validated in a larger population sample, including healthcare professionals, health students, family members of patients diagnosed with dementia and the general population [18]. Therefore, the results suggest that the DKAS-S can be useful as a generalized measure of dementia knowledge in diverse populations.

One of the main interests in developing this kind of tool is its use in assessing the effectiveness of educational interventions; in this way, we can adjust psychosocial programmes targeted towards the general population as well as towards caregivers and healthcare professionals. Adequate knowledge about dementia among healthcare staff is important to the quality of care delivered to this vulnerable population. Many studies have focused on the level of education that general practitioners and caregivers have, and some of them have focused on the effectiveness of educational programmes. However, most of them did not use standardized tools to assess levels of knowledge. In general, it has been shown that educational meetings alone or combined with other interventions can improve professional practices and healthcare outcomes for patients [30]. According to a systematic review, specific types of psychoeducation for caregivers regarding the management of neuropsychiatric symptoms were effective treatments [31]. After measuring general practitioners’ knowledge of, confidence with and attitudes towards the diagnosis and management of dementia in primary care, Turner et al. demonstrated that educational support should concentrate on epidemiological knowledge [7]. In another study, the authors examined the degree of knowledge and confidence of nursing and care assistant staff in caring for people with dementia and tried to identify factors that may contribute to greater confidence. They revealed that although staff knowledge of dementia was reasonable, confidence in addressing related situations was lower, so they concluded that training could positively influence staff confidence in addressing behaviour associated with the condition [31]. In China, community health professionals showed generally positive attitudes towards people with dementia, but they demonstrated poor dementia knowledge [32]. Therefore, the need for more educational interventions focused on dementias is widely proven for professionals, caregivers and the general population. To measure the effect of these interventions, tools such as the DKAS-S are needed.

Concerning the potential limitations of our research, the main limitation was that four items did not fit the confirmatory factory analysis. Although it can be said that the DKAS-S did not support potential subscales in same way that the original scale does, the results supported that the validation had good psychometric properties, so it could be used as a summative measure with item-level analysis. Additionally, the DKAS-S should be tested in the general population. No information about potential differences between gender in non-professional caregivers was obtained. Given that daughters are often the non-professional caregivers of LOAD, it would be interesting to know if there are differences in the knowledge of the disease between sons and daughters. However, our investigation also has some strengths. It is based on a large sample, including different cohorts from different regions of Spain. Furthermore, we have proven that the DKAS-S is a useful tool to assess the improvement of knowledge when psychoeducation for dementia or AD is provided.

## Conclusions

In conclusion, the 25-item DKAS-S is an adequate and useful instrument to measure levels of knowledge about dementia among professional and non-professional caregivers and students. The DKAS-S is a reliable and valid measure with good psychometric properties. Dementia knowledge measures are important tools to assess and improve educational interventions.

## Supplementary Information

**Additional file 1.** Rowdata of the sample

## Data Availability

The data reported in this manuscript are available in additional supporting files.

## Abbreviation

AD: Alzheimer’s disease
DKAS-S: Spanish dementia knowledge assessment scale
DQ: Dementia quiz
KAML-C: Knowledge of aging and memory loss and care
ADKS: Alzheimer’s disease knowledge scale
DKAS: Dementia knowledge assessment scale
EOAD: Early-onset AD
LOAD: Late-onset AD
CFA: Confirmatory factor analysis
DKAT2: Dementia knowledge assessment tool 2
